# Suprasternal ascending or descending aortic velocity peak variability assessment to predict fluid-responsiveness in healthy volunteers: the SADAVA-V pilot prospective study

**DOI:** 10.1007/s40477-025-01074-z

**Published:** 2025-09-18

**Authors:** Filippo Sanfilippo, Cristina Santonocito, Mateusz Zawadka, Alessandro Caruso, Giovanna Bonelli, Siddharth Dugar, Philippe Vignon, Alberto Noto

**Affiliations:** 1 Department of Anaesthesia and Intensive Care, A.O.U. Policlinico-San Marco, Site “Policlinico G. Rodolico”, Via S. Sofia N 78, 95123 Catania, Italy; 2 Department of Anaesthesiology and Intensive Care, Medical University of Warsaw, Warsaw, Poland; 3 Department of Critical Care Medicine, Respiratory Institute, Cleveland Clinic, Cleveland, OH USA; 4 Cleveland Clinic Lerner College of Medicine, Cleveland, OH USA; 5 Medical-Surgical ICU and Inserm CIC 1439, Dupuytren Teaching Hospital, Limoges, France; 6 Department of Human Pathology of Adult and Childhood “Gaetano Barresi”, University of Messina, Messina, Italy; 7 Division of Anesthesia and Intensive Care, Policlinico “G. Martino”, Messina, Italy

**Keywords:** Echocardiography, Fluid responsiveness, Velocity Time Integral, Critical care, Intensive care

## Abstract

**Aim:**

Evaluating fluid responsiveness (FR) is crucial in managing critically ill patients. Measurement of respiratory variations of blood flow (V_peak_) is physiologically sound, but blood flow sampling through the aortic valve (AV-V_peak_) is not always feasible. We assessed the feasibility of suprasternal V_peak_ (SS-V_peak_), at ascending or descending aorta level, as alternative to AV-V_peak_.

**Methods:**

Observational prospective study in spontaneously breathing healthy volunteers. We report the overall feasibility of AV- and SS-V_peak_, and calculated their interchangeability, the mean bias with limits of agreement (LoA) and percentage error (PE). We defined FR as a 10% increase in cardiac output measured non-invasively with finger-cuff method after passive leg raising.

**Results:**

We enrolled 67 volunteers; SS-V_peak_ was feasible in 65 volunteers (97%), with sampling in the ascending and descending aorta in 22/65 (33.8%) and 43/65 (66.2%) volunteers, respectively. AV-V_peak_ was feasible in 64 volunteers (95.5%). When both V_peak_ were obtained (*n* = 62), interchangeability using a 12% cut-off was 67.7% (poor agreement with kappa coefficient 0.19 [-0.02;0.41]). Clinical concordance at ascending aorta level was non-significantly higher (16/22, 73% vs 26/40, 65%; *p* = 0.583). Prediction of FR with SS-V_peak_ using the 12% cut-off was poor: sensitivity 85%; specificity 9%; positive predictive value 82%; negative predictive value 11%. Bland–Altman’s analysis revealed a mean bias -2.6% [-4.3%;-1.0%] with LoA ranging from -15.2% [− 18.1%;− 12.4%] to 10.0% [7.2%;12.8%]. The mean PE was 7.87%.

**Conclusions:**

We report excellent feasibility for SS-V_peak_, though with moderate interchangeability and accuracy; however, we found poor precision and poor performances in predicting FR in healthy volunteers.

**Supplementary Information:**

The online version contains supplementary material available at 10.1007/s40477-025-01074-z.

## Introduction

Growing evidence supports the adoption of customized fluid management strategies in intensive care unit (ICU) patients [1, 2]. Positive fluid balance is associated with worse outcomes, and fluids should be administered as any other drugs with clear indications [3–5]. As repeated fluid administration may lead to positive fluid balance, several methods have been validated to predict fluid responsiveness (FR). Each test for the anticipation of FR has its own drawbacks, and the search for tests with high feasibility at the bedspace continues.

The growth in the use of echocardiography in the ICU and, more in general, of point of care ultrasound (POCUS) [6–9] encourages to explore new non-invasive tests, especially those with high feasibility at bedspace; moreover, tests that could be also be outside the ICU (i.e. in the Emergency Department) and in spontaneously breathing patients. In this regard, the assessment of the respiratory changes in the diameter of the inferior vena cava (IVC) has been the most studied POCUS target, both in mechanically ventilated [10–12] and in spontaneously breathing patients [13–15], but it has become apparent that its use has significant drawbacks in predicting FR [16]. Conversely, the respiratory changes in blood flow measured at the heart may be more reliable as directly reflecting variations in cardiac output (CO) and stroke volume (SV) and has been validated in mechanically ventilated patients [17, 18], but not yet in spontaneously breathing ones. The respiratory variation of the peak velocity of blood flow through the aortic valve (AV-V_peak_) is relatively easy to obtain, and it has been validated in predicting FR in patients undergoing positive pressure ventilation [17, 18]. Nonetheless, a substantial proportion of ICU patients do not have adequate apical 5-chamber window for estimating AV-V_peak_, due to body habitus, lung inflation due to positive pressure ventilation, wound dressing or other factors. This has led to the investigation of parameters describing changes in forward flow at arterial level, with evaluation for instance in carotid and femoral flow [19, 20]. However, a more physiologically alternative would be the evaluation of respiratory changes in forward flow at the level of the proximal aorta, which theoretically should have a greater correlation to the variations of forward flow at aortic valve level.

As the ascending aorta, the aortic arch and/or the descending aorta could be visualized with POCUS from the suprasternal window, we designed a pilot prospective single-center study in spontaneously breathing healthy volunteers to investigate the feasibility and diagnostic accuracy in predicting fluid responsiveness of the respiratory variation of the peak velocity of blood flow through the aorta in suprasternal window (SS-V_peak_) compared to the conventional AV-V_peak_, evaluating their interchangeability. We hypothesized an excellent feasibility with good interchangeability, precision and accuracy of the values obtained suprasternal flow variation.

## Materials and methods

This is a prospective single-center study conducted on healthy volunteers and approved by the local Ethical Committee (reference protocol: 10/2023-CL-PAR). Healthy volunteers were enrolled primarily from the staffing of the Anesthesia and Critical Care Department of the “Policlinico G. Rodolico-San Marco” University Hospital; later, after amendment approved by the Ethical Committee (reference protocol: 05/2024-ES-PAR) we secondarily recruited volunteers from Medical Students of the University of Catania.

**Fig. 1:**
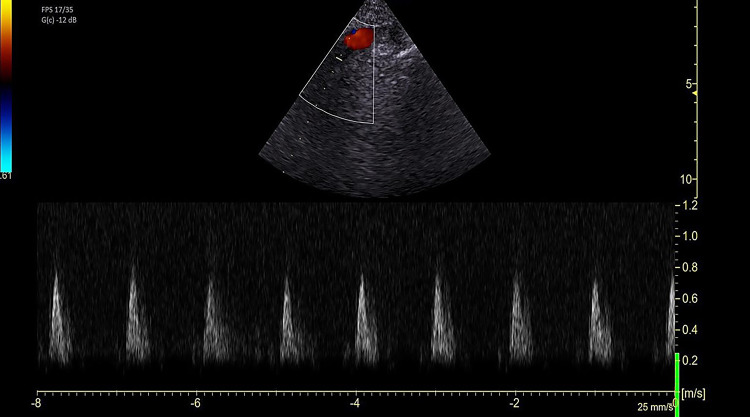
Example of peak velocity of aortic blood flow (V_peak_) sampled at the ascending aorta site. Images are taken with the aid of color doppler to recognize blood flow and ensure the best alignment

**Fig. 2:**
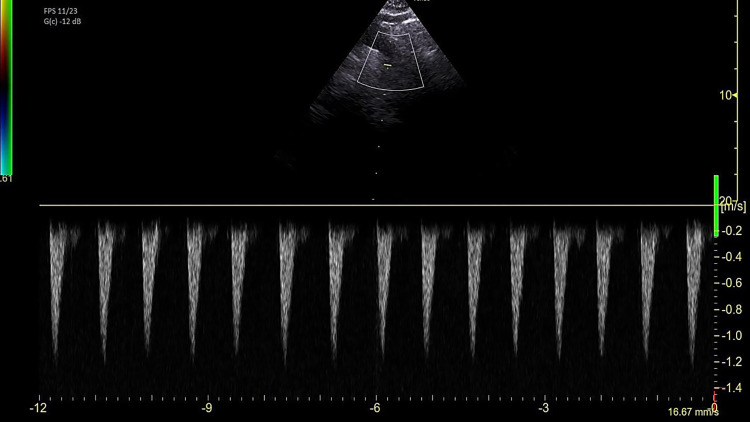
Example of peak velocity of aortic blood flow (V_peak_) sampled at the descending aorta site. Images are taken with the aid of color doppler to recognize blood flow and ensure the best alignment

In this study we focused on the AV-V_peak_ measured with Continuous Wave (CW) Doppler in the apical 5 chamber view and in the suprasternal V_peak_ SS-V_peak_; in the latter case, the aortic flow was measured at the level of the Ascending or Descending Aorta (Fig. 1 and Fig. 2, respectively). The formula used to calculate both AV-V_peak_ and the SS-V_peak_ across the respiratory cycle was: ***V***_***peak***_*** (%): [(Highest velocity – Lowest velocity) / Mean Velocity]* × *100.***

During the entire study, all volunteers were monitored using a finger-cuff non-invasive continuous cardiac output (CO) monitoring (ClearSight®, Edwards Lifesciences Corp, Irvine, CA, USA) system for advanced hemodynamic parameters. Data in this study were recorded and reported according to the PRICES guidelines [21, 22] with checklist as per FR studies (Supplementary material).

### Study procedures

**Fig. 3:**
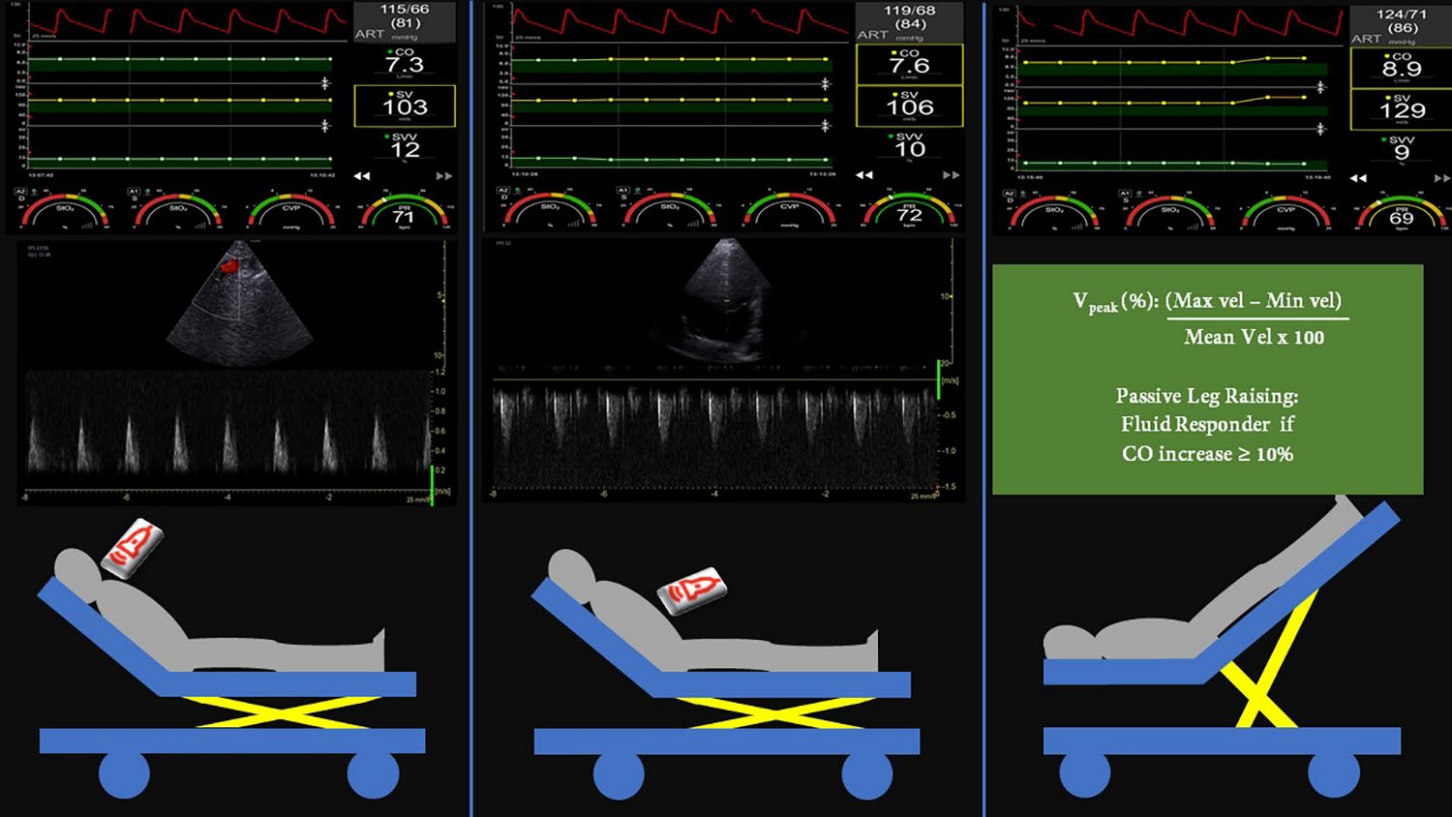
Positioning of the healthy volunteer and measurements. **A** and **B**: Semi-recumbent position used during the acquisition of images from the suprasternal **A** and the apical **B** window to obtain the respiratory variation of the peak velocity of blood flow (V_peak_). During the echocardiographic measurement, hemodynamic conditions were recorded from a finger-cuff non-invasive continuous cardiac output (CO) monitoring device. **C**: Participant’s position is changed with a passive leg raising maneuver, the hemodynamic changes recorded and later compared to the baseline values. ART: arterial pressure values; *PR* pulse rate (heart rate), *SV*: stroke volume, *SVV* SV variation

All volunteers were examined in standardized semi-recumbent position during quiet and regular breathing and were in sinus rhythm. The ultrasonographic assessment was conducted using a General Electric (GE) Venue Go R2 ultrasound machine by two operators (MZ and FS). Both operators first conducted each echocardiographic exam initially ruling out the presence of at least moderate systolic dysfunction and/or at least moderate valve disease. Once this had been accomplished, the study started and the entire procedure with its three steps is shown in Fig. 3.

- The operator calculated the SS-V_peak_ (%) at the level of the Ascending and/or Descending Aorta site through the suprasternal window (Fig. 3A). Two independent investigators recorded the baseline values of CO, SV and heart rate (HR) provided by the non-invasive continuous monitoring with finger-cuff method. The operator recorded the Continuous Wave Doppler signal in the Ascending or Descending Aorta selecting the one with best alignment (seeking Doppler angle closest to 0°). If both sites offered a good alignment, the operator recorded the SS-V_peak_ at the Ascending Aorta site (closest anatomical site to aortic valve).
- The operator obtained the AV-V_peak_ (%) from an apical 5-chamber view (Fig. 3B). Two independent investigators registered the baseline values of CO, SV and HR at image acquisition.
- After the measurements of AV-V_peak_ and SS-V_peak_ were accomplished, volunteers passively received a PLR maneuver[23] (Fig. 3C), and again hemodynamic values were recorded following the same sequence.

For each V_peak_ measurement, we calculated the *Δ*CO defined as the difference between the baseline CO recorded at the time of sampling AV-V_peak_ and SS-V_peak_, and the highest value of CO recorded within the first 5 min of PLR. Volunteers were defined as FR if their CO increased by at least 10%. We also recorded the time occurring from the begin of the PLR maneuver to the highest observed CO value. The calculations for the two V_peak_ values were performed off-line and weeks apart from the image recording to avoid interpretation bias related to the hemodynamic response to PLR.

### Statistical analysis

Our study is a feasibility pilot investigation as the AV-V_peak_ had been validated in mechanically ventilated patients [18], but not in spontaneously breathing individuals. As the study enrolled healthy volunteers, we estimated that at least 75% of them would be FR after PLR [24].

The sample size was estimated according to an expected area under the curve of 0.80 with a null-hypothesis of 0.50, a statistical power set at 80% and an alpha level at 0.05. We obtained a sample size calculation of 52 cases. However, considering volunteers with suboptimal windows for AV- and SS-V_peak_, as well as the chance of FR participants above the estimated one (75%), we preventively planned to increase the sample size by 25% (*n* = 65).

There exist no prior study evaluating the interchangeability of AV-V_peak_ and SS-V_peak_, hence a sample size calculation for this aspect of the study could not be ascertained. As mentioned above, no prior literature exists no cutoff for SS-V_peak_ to determine FR; hence, the cutoff of 12%, which was previously validated for AV-V_peak_ albeit in mechanically ventilated patients, was agreed [17]. The 12% cutoff was also set for the graphical evaluation of the interchangeability. Hence, each time the SS-V_peak_ fell into the same category as the AV-V_peak_ (≥ 12% or < 12%), we assigned a good clinical concordance, poor concordance otherwise. Using these definitions, we computed the kappa coefficient and plotted a clinical concordance plot.

After PLR maneuver we used a 10% increase in CO to differentiate FR and non-FR; accordingly, we calculated sensitivity and specificity of the SS-V_peak_ and AV-V_peak_ to discriminate the FR, as well as their positive and negative predictive values (PPV and NPV, respectively). The precision and accuracy of the SS-V_peak_ were derived from the Bland and Altman analysis providing not only a graphical representation of the agreement but also an objective measure of the mean bias (average of the differences) between methods (accuracy), and the width of the limits of agreement (LoA; describing the precision). Mean bias and LoA are described with their 95% confidence interval (CI). Moreover, the precision of the agreement is reported in terms of percentage error (PE) and calculated as follow [25]:

$$Percentage error={t}_{\alpha ,n-1}\times \frac{SD}{mean}$$

where n represents the sample size; mean and SD the mean and standard deviation of all the differences between the two methods, respectively; and ${t}_{\alpha ,n-1}$ the t-value of the Student law corresponding to n-1 degrees of freedom and a type I error α (here set at 0.05). The above analyses were performed using R (R version 4.3.3) and JASP (JASP Team – version 0.18.3). Statistics was conducted with blinding from site of V_peak_ sampling.

## Results

### Descriptive and Feasibility analysis

**Fig. 4:**
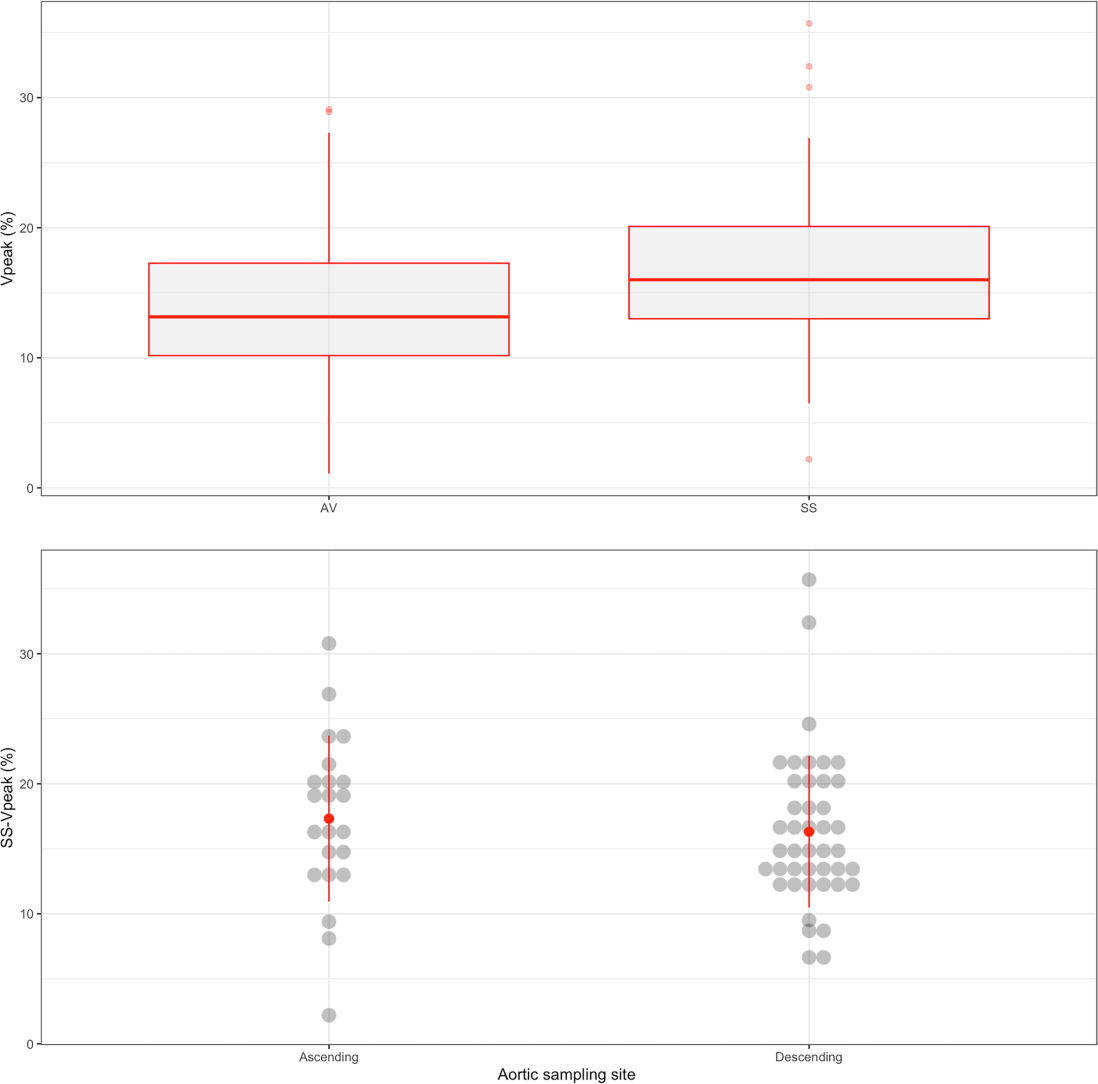
Top: Boxplots of the baseline values of the respiratory variation of the peak velocity of blood flow obtained through the aortic valve (AV-V_peak_) and in suprasternal window (SS-V_peak_). Bottom: Dotplots of the SS-V_peak_ baseline values provided according to the anatomical region of sampling (ascending or descending aorta)

We conducted the study over 5 months, enrolling 67 volunteers (*n* = 45 males, 67%); their age, weight and height were 33 ± 9 years, 74 ± 13 kg and 172 ± 10 cm, respectively. The AV-V_peak_ was obtained in 64/67 participants (95.5%), and the SS-V_peak_ was recorded in 65/67 volunteers (97%). Regarding the site of sampling for the SS-V_peak_, in 22 cases (33.8%) it was sampled at the ascending aorta, and in the remaining 43 cases (66.2%) the operator preferred to sample the descending aorta flow (better alignment of the Doppler flow). As shown in Fig. 4a, the overall SS-V_peak_ values were significantly higher than overall AV-V_peak_ data (16.7 ± 6.0% vs 13.9 ± 6.0%, *p* = 0.002). Regarding the SS-V_peak_, its values sampled at the ascending aorta site (*n* = 22, 17.2 ± 6.5%) were not significantly different from to those gathered at the descending aorta level (*n* = 43, 16.3 ± 4.8%; *p* = 0.289; Fig. 4b).

Table 1 reports the values of CO, SV, and HR recorded with finger-cuff non-invasive continuous monitoring during the acquisition of AV- and SS-V_peak_. These values were not significantly different, although a trend was seen for a higher CO during AV-V_peak_ sampling (*p* = 0.068). The maximal values of CO, SV and HR obtained after PLR and the mean change from their respective baseline values (measurement of SS-V_peak_ and AV-V_peak_, respectively) are also reported in Table 1. The mean time took to reach the highest increment of CO after PLR was 22.7 ± 8.7 s.

**Table 1:** Values of cardiac output (CO, L/min), stroke volume (SV, ml) and heart rate (HR, beats/min) measured continuously with finger-cuff method in healthy volunteers during the phases of the study

	SS-V_peak_ (*n* = 65)	AV-V_peak_ (*n* = 64)	*p*	After PLR	ΔPLR (%) SS	ΔPLR (%) AV
CO (L/min)	6.4 ± 1.6	6.6 ± 1.6	0.068	8.0 ± 1.8	26.1 ± 20.3	23.1 ± 18.1
SV (ml)	92.1 ± 18.3	94.6 ± 18.4	0.179	103.2 ± 18.6	13.8 ± 15.3	11.7 ± 12.4
HR (bpm)	71.6 ± 11	71.6 ± 10.3	0.450	75.8 ± 11.2	6.7 ± 13.1	5.8 ± 11.9

Values are reported at baseline, hence when the respiratory variations of the peak velocity of blood flow were measured at Suprasternal Aorta and at apical 5-chambers Aortic Valve sites (SS-V_peak_ and AV-V_peak_, respectively), and subsequently after a Passive Leg Raising (PLR) maneuver. Accordingly, we describe the relative changes after PLR from baseline (%). Data are reported as mean and standard deviation

The baseline values of CO, SV and HR in the subgroups of SS-V_peak_ according to the site of sampling (ascending, *n* = 22) or descending aorta (*n* = 43) are provided as Supplementary material, and were not statistically different.

### ***Interchangeability and agreement of V***_***peak***_*** values***

**Fig. 5:**
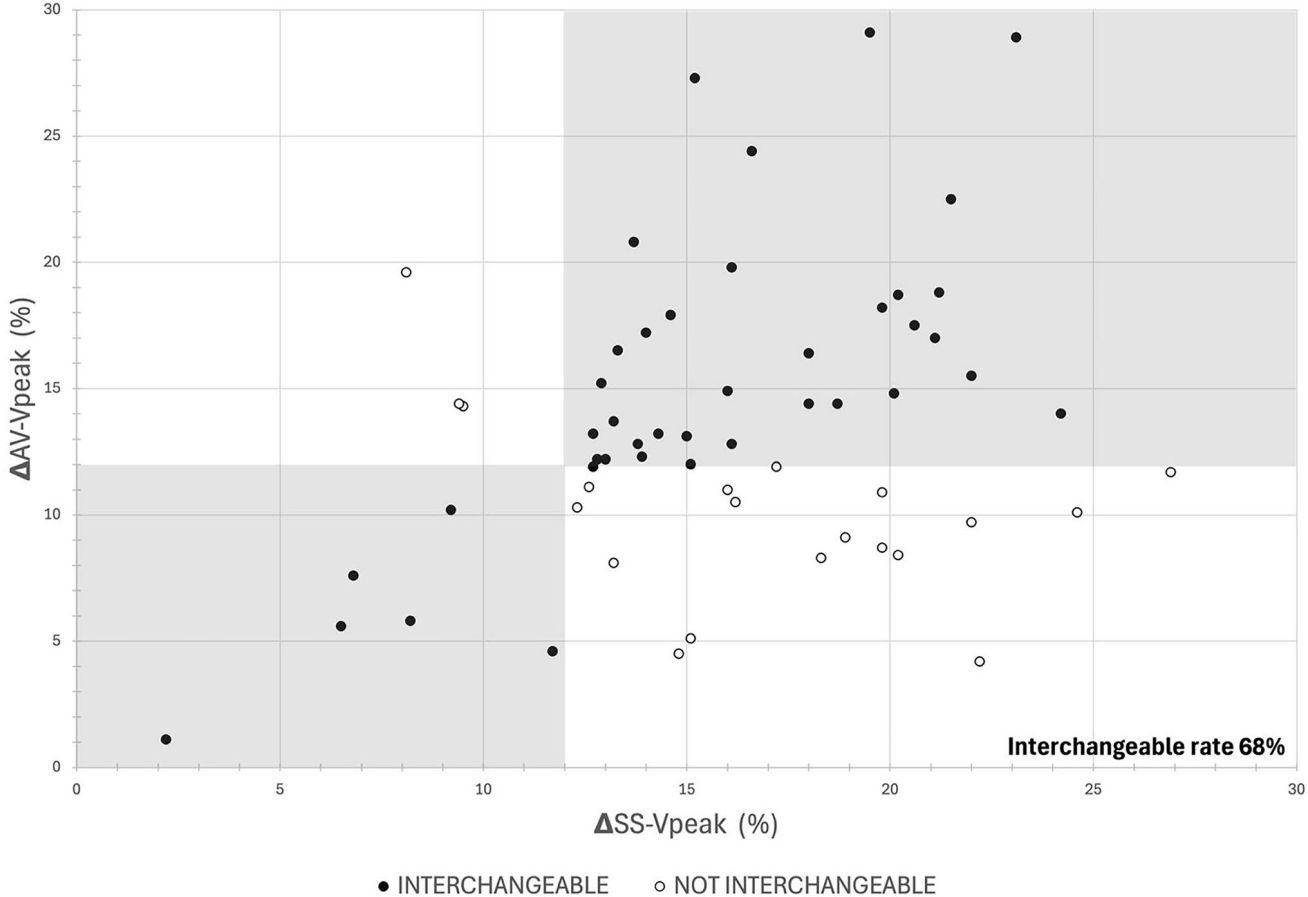
Graphical representation of interchangeability between measurements of peak velocity of blood flow measured at Suprasternal and Aortic Valve sites (SS-V_peak_ and AV-V_peak_, respectively) according to a cut-off of 12%

In 62 volunteers (92.5%), we obtained both AV-V_peak_ and SS-V_peak_. As shown in Fig. 5, the proportion of volunteers in whom the two V_peak_ values were in clinical concordance (using a cut-off of 12%, grey areas) was 42 out of 62, with 36 showing both V_peak_ values above and 6 with both V_peak_ values reported below the cut-off. In the remaining 20 cases, we observed the majority (*n* = 17) with AV-V_peak_ below the cut-off whilst SS-V_peak_ value was higher than 12%. In three cases, we detected a discordance with AV-V_peak_ above and SS-V_peak_ below the cut-off. Overall, these findings reflect an interchangeability rate of 67.7%. The kappa coefficient was computed at 0.19 (95% CI, -0.02; 0.41), corresponding to a poor agreement. Dotplots of the recorded values of AV- and SS-V_peak_ according to FR are provided in Supplementary material.

A subgroup analysis showed non-significant higher clinical concordance for the SS-V_peak_ sampled at the ascending aorta level (16/22, 73%) as compared to the subgroup of SS-V_peak_ obtained at the descending aorta site (n = 26/40, 65%; *p* = 0.583).

### Prediction of fluid responsiveness

As shown in Table 2, according to the changes in CO recorded after the PLR maneuver, 53 out of 64 volunteers with AV-V_peak_ available were classified as FR (82.8%), and the remaining 11 as non-FR (17.2%). In the 65 volunteers where we measured the SS-V_peak_, 54 were classified as FR (83.1%), and 11 were not (16.9%). Mean values of AV-V_peak_ and SS-V_peak_ in FR and non-FR are also shown. The prediction of FR with an AV-V_peak_ ≥ 12% had the following characteristics: sensitivity 68%; specificity 75%; PPV 92%; NPV 35%. The prediction of FR with the SS-V_peak_ using the same cut-off was weaker: sensitivity 85%; specificity 9%; PPV 82%; NPV 11%.

**Table 2:** Healthy volunteers divided according to fluid responsiveness after passive leg raising (PLR)

Fluid responsiveness after PLR		ΔCO < 10%	ΔCO ≥ 10%
SS-V_peak_ (*n* = 65)	*n *=	11 (16.9%)	54 (83.1%)
	V_peak_	17.4 ± 6.3%	16.5 ± 5.8%
AV-V _peak_ (*n* = 64)	*n* =	11 (17.2%)	53 (82.8%)
	V_peak_	8.9 ± 5.3%	14.9 ± 5.6%

Respiratory variations of the peak velocity (V_peak_) according the site of recording is shown the two sites, the Suprasternal and the Aortic Valve acoustic windows (SS-V_peak_ and AV-V_peak_, respectively). Values are reported as numbers and percentages, and as mean and standard deviation. Fluid responsiveness was defined according to the change in cardiac output (CO, L/min) after PLR maneuver from the baseline value recorded during acquisition of V_peak_

### Precision, accuracy and PE

**Fig. 6:**
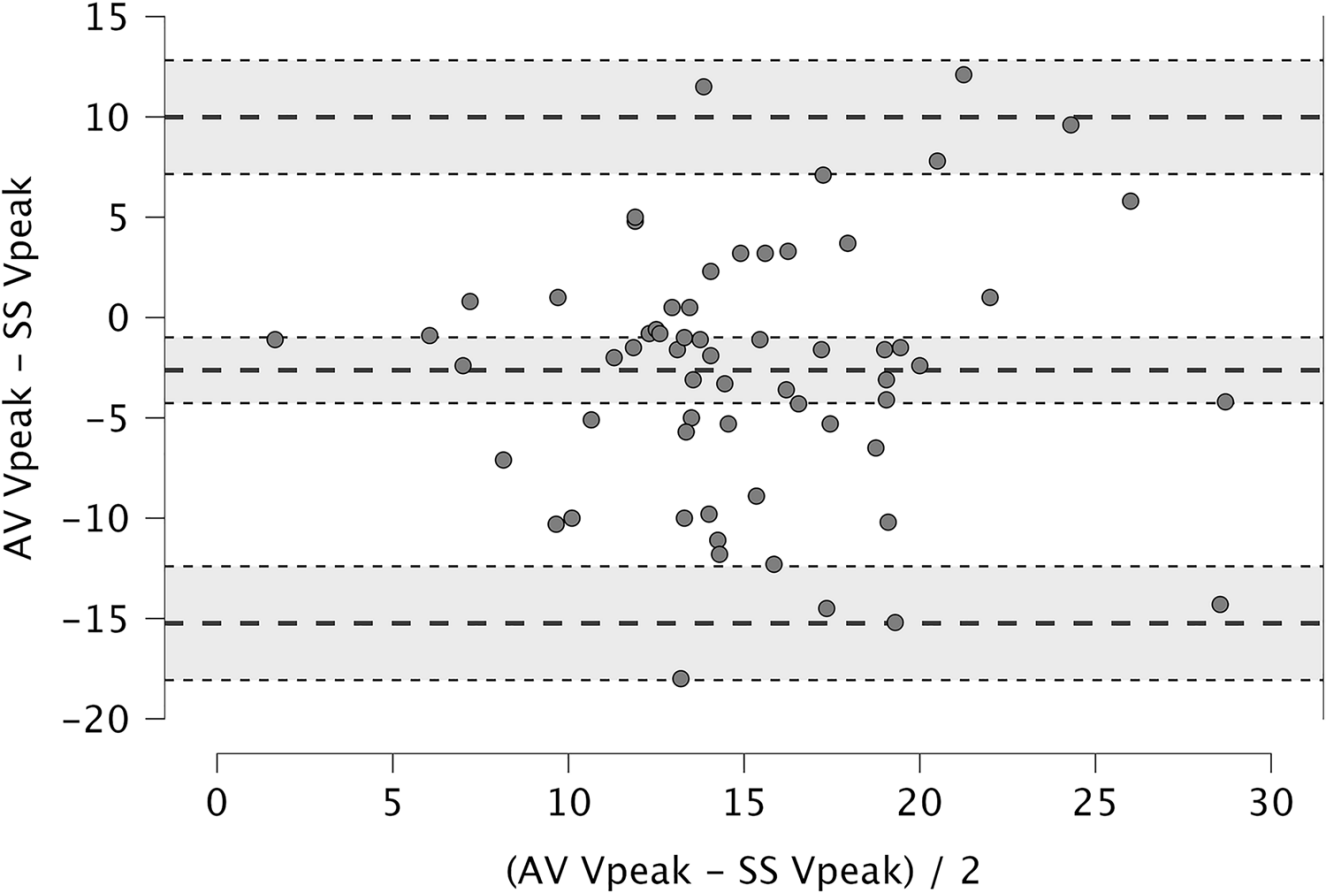
Bland–Altman plot for the peak velocity of blood flow measured at Suprasternal and Aortic Valve sites (SS-V_peak_ and AV-V_peak_, respectively)

The Shapiro–Wilk test and the graphical visualization were considered to assess the normality of distribution of the differences between the two methods. The Bland and Altman’s analysis revealed a mean bias of − 2.6% (95%CI − 4.3%; − 1.0%), suggesting overestimation of the SS-V_peak_ as compared to the AV-V_peak_; the LoA ranged from − 15.2% (95%CI − 18.1%; − 12.4%) to 10.0% (95% CI 7.2%; 12.8%; Fig. 6). The mean PE was 7.87%.

## Discussion

We conducted a technical pilot prospective study in healthy volunteers to determine the feasibility of obtaining the respiratory variation of the peak velocity of the aortic flow obtained through the suprasternal acoustic window (SS-V_peak_), sampled either in the ascending or descending aorta. We further evaluated the concordance of the respiratory variations of the SS-V_peak_ and the peak velocity obtained through the apical 5-chambers view (AV-V_peak_), as well as the accuracy and precision of the measurements.

Overall, we found an excellent feasibility of the SS-V_peak_, obtained in 65 out of 67 participants (97%). Such result is theoretically promising as it substantiates the availability of another site to measure respiratory changes in blood flow, with sampling obtained closer to the heart as compared to flow sampled in the carotid, brachial, radial or femoral artery. However, the poor specificity of SS-V_peak_ taken together with the moderate interchangeability and the poor correlation with AV-V_peak_ warrant caution, severely precluding its current role in clinical decision-making. Using a cutoff of 12% change, validated only in mechanically ventilated patients, we found that around two-thirds of measurements were interchangeable with AV-V_peak_. Interestingly, the subgroup analysis according to the site of sampling (ascending or descending aorta) showed greater interchangeability at the more proximal site; however, the operator preferred in around two-thirds of our cases to sample the flow in the descending aorta as it provided a better Doppler alignment. We believe that future studies on mechanically ventilated patients studying the feasibility and the predictive values of the SS-V_peak_ should consider focusing on the Doppler sampling at the ascending aorta level. Indeed, interchangeability with the reference measurement (i.e., AV-V_peak_) is more relevant than the accuracy of absolute value of this hemodynamic parameter since the potential underestimation related to suboptimal Doppler beam alignment with forward flow applies on both the end-expiratory and end-inspiratory maximal velocities, hence fails to alter the resulting ratio. Future studies are needed before considering to open the door to the SS-V_peak_, which could become helpful when the evaluation at the level of the left ventricular outflow tract cannot be achieved due to poor acoustic windows and/or poor alignment, a condition frequently encountered in mechanically ventilated patients. Moreover, the utility of SS-V_peak_ outside the ICU settings might be of special interest, particularly in the operating room where the evaluation of FR with most POCUS methods is technically not feasible. Indeed, with the exception of some specialties with the surgical field located in the head-neck region, the calculation of SS-V_peak_ may be accessible in most of surgical specialties (e.g., abdominal, thoracic, orthopedic and vascular). As long as there is no evidence of AV stenosis, the AV-V_peak_ is influenced by the heart–lung interaction similar to the respiratory variation of pulse pressure and/or SV. Under steady breathing pattern, changes in blood flow sampled at the heart level are reflected distally. Hence, we hypothesized that the closest the sampling to the heart, the greater should be the correlation between changes in the measured flows and velocities. Such hypothesis was partially confirmed by the greater interchangeability of SS-V_peak_ obtained at the ascending aorta level. In line with our hypothesis, in trauma patients with shock the V_peak_ measured in the carotid or brachial artery showed slightly higher performances in predicting FR as compared to its value gathered in the femoral artery [26]. Of note, from technical perspectives and contrary to the sampling at the level of carotid, brachial or femoral arteries, the evaluation of blood flow and/or velocity in the ascending or descending aorta does not require an angle correction of the pulsed wave Doppler. Despite our hypothesis of sampling respiratory variability of blood flow/velocity in the aorta to be physiologically sound, we found a poor correlation between the two V_peak_ measures (kappa coefficient 0.19). The mean bias was relatively low (-2.6%, suggesting small overestimation of SS-V_peak_) though with a wide LoA and low precision, suggesting that in spontaneously breathing subjects the two assessments are not interchangeable.

In our study, 80% of our volunteers were found to be FR based on changes in CO measured with the finger cuff method after a PLR maneuver. Comparing the two V_peak_ methods for the prediction of FR, we found that SS-V_peak_ had much lower predictivity, particularly had a very low specificity (9%). However, this analysis was performed using the cut-off of 12% which has not been validated in spontaneously breathing patients. Several considerations should be made to explain our findings. As first consideration, the heart–lung interaction is much less pronounced during spontaneous quiet breathing than under positive-pressure ventilation, and may confound results. Secondly, the high aortic compliance of young and healthy volunteers is likely to further blur the respiratory-induced variation of blood flow velocity [27, 28], and results could have been different in elderly patients or in those with comorbidities. This may account for the differences observed between the ascending and descending aorta. Third, our results seem influenced by the site of sampling, with the descending aorta providing better alignment in most cases but showing poorer clinical concordance with the AV-V_peak_ and worse performances in the prediction of FR. In general, as the possibility to obtain a good apical 5-chamber view is often limited in mechanically ventilated patients [29], other methods should be sought. The inferior vena cava has been intensely studied but its use is subject to numerous limitations [16], and visualization can be sometimes challenging [30]. Considering such limitations, the evaluation of arterial blood flow and velocities may represent a more effective target. Hence, it seems sound to direct scientific efforts towards the study of changes in blood flow more peripheral to the heart. In this regard, the most studied anatomical sites have been the carotid and the femoral artery. A recent meta-analysis evaluating the role of several parameters gathered from carotid Doppler signal in predicting FR has questioned their clinical value with limited ability in predicting FR. Nonetheless, among all carotid Doppler variables considered, the change in carotid Doppler peak velocity (carotid-V_peak_) showed the best performance with an AUC of 0.82, performing better than the carotid blood flow [20]. The femoral artery has been studied for the same purpose. Despite promising experience by Preau et al. in spontaneously breathing patients [31], their results were contradicted by Girotto et al. in a cohort of critically ill patients where both the carotid and femoral artery blood flow and peak velocity performed poorly in predicting FR. Moreover, the feasibility of measuring carotid blood flow was 76% and much lower in the femoral artery (27%) [19].

### Limitations

There are several limitations in this pilot study. First, the selected population of healthy volunteers is likely to influence the results in several respects, including the feasibility, which was a primary endpoint of the study. Among others, our population mainly consisted of young subjects with normal body mass index. Though the SS window should not be largely affected by an increased weight, it is not possible to exclude a lower feasibility in obese patients and in those undergoing mechanical ventilation with some degree of lung hyperinflation. Clinical studies are needed in this regard. Second, definition of FR and its detection is probably complex in young and healthy participants as they rapidly adapt to the increase in preload produced by the PLR with an early and transient response. The mean time between initiation of PLR and the highest measured CO in our study was about 23 s, a finding similar what Monnet et al. described in ICU patients, where all responders had an increase in aortic blood flow by 30 s after PLR [32]. Interestingly, a recent study investigated FR in healthy volunteers and found that no participant exhibited a velocity time integral (VTI) increase above 7% after a change in position (from semi-recumbent to supine) or after fluid loading. Of note, in this study the echocardiographic measurements were taken 1 min after the intervention [33], so possibly after the peak of variation. This could at least partially explain their results, highlighting the complexity of assessing FR in healthy and young volunteers. In this regard, we picked-up the highest increase in CO after PLR measured continuously with the non-invasive finger-cuff method. Third, we used non-calibrated system to monitor CO, which is an additional limitation; however, this method allowed us a continuous monitoring to detect the greatest CO change. Fourth, from echocardiographic perspectives, the use of CW for the estimation of peak velocity could be seen as a limitation as compared to the trace of VTI with pulse wave Doppler. We used the CW Doppler because measuring the peak velocity is easier and faster than calculating a VTI (which requires tracing of Doppler envelope), hence our approach could increase the clinical feasibility. Moreover, it could have less intra- and inter-operator variability. Further, to increase the feasibility, we decided to include measurements in both thoracic aorta sites (ascending or descending), giving preference according to the best Doppler alignment. It should be also considered that in a population of healthy volunteers (normal AV morphology and function, no left ventricular outflow tract obstruction), the CW (and consequently peak velocity) should be a good and reliable alternative to tracing the VTI, since the evaluation of respiratory changes of aortic peak velocity (V_peak_) has strong correlation with SV changes for FR prediction [34]. Fifth, we studied the interchangeability of the AV- and SS-V_peak_ values based on a cut-off validated for mechanically ventilated patients, as no threshold has been studied for spontaneously breathing patients [17].

## Conclusions

In a population of healthy volunteers, we report an excellent feasibility (97%) for the SS-V_peak_,. However, the SS-V_peak_ showed only moderate interchangeability and accuracy with the AV-V_peak_, along with poor precision and concordance. In general, it had poor performances in predicting fluid responsiveness. Further studies are recommended in sedated and mechanically ventilated patients to confirm such high feasibility of SS-V_peak_ and to evaluate its clinical performances in predicting fluid responsiveness.

**Take home message** The measurement of respiratory variations of blood flow (V_peak_) can be used for the evaluation of fluid responsiveness in critically ill patients, but blood flow sampling through the aortic valve is not always feasible. In a population of spontaneously breathing healthy volunteers, we have shown a high feasibility of the V_peak_ obtained in suprasternal window at ascending or descending aorta level, though this parameter had only moderate interchangeability with aortic valve V_peak_.

## Supplementary Information

Below is the link to the electronic supplementary material.

Supplementary file1 (TIFF 16387 KB)

Supplementary file2 (DOCX 1712 KB)

Supplementary file3 (DOCX 14 KB)

## Data Availability

Data available from corresponding author FS at reasonable request.

## References

[1] Muñoz F, Born P, Bruna M, Ulloa R, González C, Philp V et al (2024) Coexistence of a fluid responsive state and venous congestion signals in critically ill patients: a multicenter observational proof-of-concept study. Critical care (London, England) 28:52 38374167 10.1186/s13054-024-04834-1 PMC10877871

[2] Kattan E, Castro R, Miralles-Aguiar F, Hernández G, Rola P (2022) The emerging concept of fluid tolerance: a position paper. J Crit Care 71:154070 35660844 10.1016/j.jcrc.2022.154070

[3] Malbrain M, Martin G, Ostermann M (2022) Everything you need to know about deresuscitation. Intensive Care Med 48:1781–1786 35932335 10.1007/s00134-022-06761-7 PMC9362613

[4] Messmer AS, Zingg C, Müller M, Gerber JL, Schefold JC, Pfortmueller CA (2020) Fluid overload and mortality in adult critical care patients-a systematic review and meta-analysis of observational studies. Crit Care Med 48:1862–1870 33009098 10.1097/CCM.0000000000004617

[5] Malbrain M, Van Regenmortel N, Saugel B, De Tavernier B, Van Gaal PJ, Joannes-Boyau O et al (2018) Principles of fluid management and stewardship in septic shock: it is time to consider the four D’s and the four phases of fluid therapy. Ann Intensive Care 8:66 29789983 10.1186/s13613-018-0402-x PMC5964054

[6] Vieillard-Baron A, Millington SJ, Sanfilippo F, Chew M, Diaz-Gomez J, McLean A et al (2019) A decade of progress in critical care echocardiography: a narrative review. Intensive Care Med 45:770–788 30911808 10.1007/s00134-019-05604-2

[7] Messina A, Robba C, Bertuetti R, Biasucci D, Corradi F, Mojoli F et al (2022) Head to toe ultrasound: a narrative review of experts’ recommendations of methodological approaches. J Anesth Analg Crit Care 2:44 37386682 10.1186/s44158-022-00072-5 PMC9589874

[8] Beaubien-Souligny W, Rola P, Haycock K, Bouchard J, Lamarche Y, Spiegel R et al (2020) Quantifying systemic congestion with point-of-care ultrasound: development of the venous excess ultrasound grading system. Ultrasound J. 10.1186/s13089-020-00163-w 32270297 10.1186/s13089-020-00163-w PMC7142196

[9] Sauza-Sosa JC, Arratia-Carlin K, Fernandez-Tapia J (2022) Point-of-care ultrasound assessment with handheld ultrasound device attached to cell phone. J Clin Ultrasound 50:284–285 34797608 10.1002/jcu.23097

[10] Barbier C, Loubières Y, Schmit C, Hayon J, Ricôme JL, Jardin F et al (2004) Respiratory changes in inferior vena cava diameter are helpful in predicting fluid responsiveness in ventilated septic patients. Intensive Care Med 30:1740–1746 15034650 10.1007/s00134-004-2259-8

[11] Sanfilippo F, La Via L, Dezio V, Amelio P, Genoese G, Franchi F et al (2023) Inferior vena cava distensibility from subcostal and trans-hepatic imaging using both M-mode or artificial intelligence: a prospective study on mechanically ventilated patients. Intensive Care Med Exp 11:40 37423948 10.1186/s40635-023-00529-z PMC10329966

[12] Zhang H, Zhang Q, Chen X, Wang X, Liu D (2019) Respiratory variations of inferior vena cava fail to predict fluid responsiveness in mechanically ventilated patients with isolated left ventricular dysfunction. Ann Intensive Care 9:113 31591663 10.1186/s13613-019-0589-5 PMC6779682

[13] Airapetian N, Maizel J, Alyamani O, Mahjoub Y, Lorne E, Levrard M et al (2015) Does inferior vena cava respiratory variability predict fluid responsiveness in spontaneously breathing patients? Critical care (London, England) 19:400 26563768 10.1186/s13054-015-1100-9 PMC4643539

[14] Muller L, Bobbia X, Toumi M, Louart G, Molinari N, Ragonnet B et al (2012) Respiratory variations of inferior vena cava diameter to predict fluid responsiveness in spontaneously breathing patients with acute circulatory failure: need for a cautious use. Crit Care 16:R188 23043910 10.1186/cc11672 PMC3682290

[15] Zawadka M, Santonocito C, Dezio V, Amelio P, Messina S, Cardia L et al (2024) Inferior vena cava distensibility during pressure support ventilation: a prospective study evaluating interchangeability of subcostal and trans-hepatic views, with both M-mode and automatic border tracing. J Clin Monit Comput. 10.1007/s10877-024-01177-8 38819726 10.1007/s10877-024-01177-8 PMC11427491

[16] Via G, Tavazzi G, Price S (2016) Ten situations where inferior vena cava ultrasound may fail to accurately predict fluid responsiveness: a physiologically based point of view. Intensive Care Med 42:1164–1167 27107754 10.1007/s00134-016-4357-9

[17] Feissel M, Michard F, Mangin I, Ruyer O, Faller JP, Teboul JL (2001) Respiratory changes in aortic blood velocity as an indicator of fluid responsiveness in ventilated patients with septic shock. Chest 119:867–873 11243970 10.1378/chest.119.3.867

[18] Vignon P, Repessé X, Bégot E, Léger J, Jacob C, Bouferrache K et al (2017) Comparison of echocardiographic indices used to predict fluid responsiveness in ventilated patients. Am J Respir Crit Care Med 195:1022–1032 27653798 10.1164/rccm.201604-0844OC

[19] Girotto V, Teboul JL, Beurton A, Galarza L, Guedj T, Richard C et al (2018) Carotid and femoral doppler do not allow the assessment of passive leg raising effects. Ann Intensive Care 8:67 29845417 10.1186/s13613-018-0413-7 PMC5975047

[20] Walker SCD, Lipszyc AC, Kilmurray M, Wilding H, Akhlaghi H (2024) Questioning the role of carotid artery ultrasound in assessing fluid responsiveness in critical illness: a systematic review and meta-analysis. Crit Care Res Pract 2024:9102961 38716052 10.1155/2024/9102961 PMC11074915

[21] Huang S, Sanfilippo F, Herpain A, Balik M, Chew M, Clau-Terré F et al (2020) Systematic review and literature appraisal on methodology of conducting and reporting critical-care echocardiography studies: a report from the European Society of Intensive Care Medicine PRICES expert panel. Intensive Care Med 10:49 10.1186/s13613-020-00662-y PMC7183522 32335780

[22] Sanfilippo F, Huang S, Herpain A, Balik M, Chew MS, Clau-Terré F et al (2021) The PRICES statement: an ESICM expert consensus on methodology for conducting and reporting critical care echocardiography research studies. Intensive Care Med 47:1–13 33275163 10.1007/s00134-020-06262-5

[23] Monnet X, Teboul JL (2015) Passive leg raising: five rules, not a drop of fluid! Critical care (London, England) 19:18 25658678 10.1186/s13054-014-0708-5 PMC4293822

[24] Godfrey GE, Dubrey SW, Handy JM (2014) A prospective observational study of stroke volume responsiveness to a passive leg raise manoeuvre in healthy non-starved volunteers as assessed by transthoracic echocardiography. Anaesthesia 69:306–313 24641636 10.1111/anae.12560

[25] Montenij LJ, Buhre WF, Jansen JR, Kruitwagen CL, de Waal EE (2016) Methodology of method comparison studies evaluating the validity of cardiac output monitors: a stepwise approach and checklist. Br J Anaesth 116:750–758 27199309 10.1093/bja/aew094

[26] Zhang Q, Shi XR, Shan Y, Wan J, Ju X, Song X et al (2021) Respiratory variations in peak peripheral artery velocities and waveforms for rapid assessment of fluid responsiveness in traumatic shock patients. Med Sci Monit 27:e928804 33414360 10.12659/MSM.928804 PMC7802376

[27] Fukuie M, Yamabe T, Kimura R, Zhu DC, Ohyama-Byun K, Maeda S et al (2024) Ascending aortic impedance in young endurance athletes: a time-resolved phase-contrast MRI study. J Appl Physiol (1985) 136:555–566 38234292 10.1152/japplphysiol.00184.2023

[28] Tarumi T, Yamabe T, Fukuie M, Kimura R, Zhu DC, Ohyama-Byun K et al (2021) Proximal aortic compliance in young male endurance athletes: an MRI study. Med Sci Sports Exerc 53:543–550 32881770 10.1249/MSS.0000000000002508

[29] Flower L, Madhivathanan PR, Andorka M, Olusanya O, Roshdy A, Sanfilippo F (2021) Getting the most from the subcostal view: the rescue window for intensivists. J Crit Care 63:202–210 32958350 10.1016/j.jcrc.2020.09.003

[30] Sanfilippo F, La Via L, Dezio V, Santonocito C, Amelio P, Genoese G et al (2023) Assessment of the inferior vena cava collapsibility from subcostal and trans-hepatic imaging using both M-mode or artificial intelligence: a prospective study on healthy volunteers. Intensive Care Med Exp 11:15 37009935 10.1186/s40635-023-00505-7 PMC10068684

[31] Préau S, Saulnier F, Dewavrin F, Durocher A, Chagnon JL (2010) Passive leg raising is predictive of fluid responsiveness in spontaneously breathing patients with severe sepsis or acute pancreatitis. Crit Care Med 38:819–825 20016380 10.1097/CCM.0b013e3181c8fe7a

[32] Monnet X, Rienzo M, Osman D, Anguel N, Richard C, Pinsky MR et al (2006) Passive leg raising predicts fluid responsiveness in the critically ill. Crit Care Med 34:1402–1407 16540963 10.1097/01.CCM.0000215453.11735.06

[33] Kim YH, Lee JH (2024) Prediction of fluid responsiveness in spontaneously breathing patients with hemodynamic stability: a prospective repeated-measures study. Sci Rep 14:14451 38914634 10.1038/s41598-024-65554-8 PMC11196262

[34] Slama M, Masson H, Teboul JL, Arnout ML, Susic D, Frohlich E et al (2002) Respiratory variations of aortic VTI: a new index of hypovolemia and fluid responsiveness. Am J Physiol Heart Circ Physiol 283:H1729–H1733 12234829 10.1152/ajpheart.00308.2002

