# Supplementary figures and images for: Suprasternal ascending or descending aortic velocity peak variability assessment to predict fluid-responsiveness in healthy volunteers: the SADAVA-V pilot prospective study
Source: J Ultrasound. 2025 Sep 18;28(4):931–41. doi: 10.1007/s40477-025-01074-z (PMC12675857; doi:10.1007/s40477-025-01074-z)

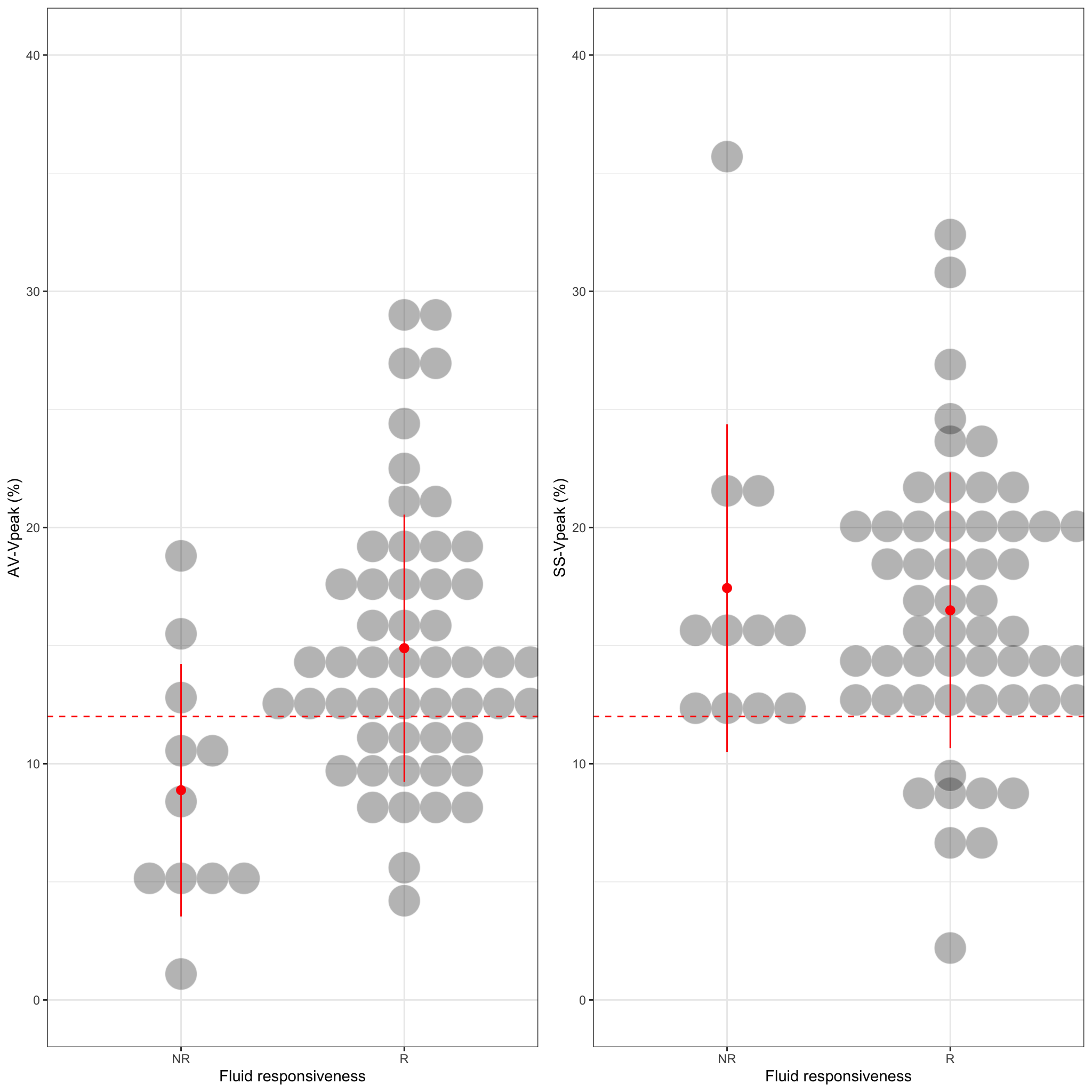

Supplement: Supplementary file 1 — Supplementary file1 (TIFF 16387 KB) [file 40477_2025_1074_MOESM1_ESM.tiff]

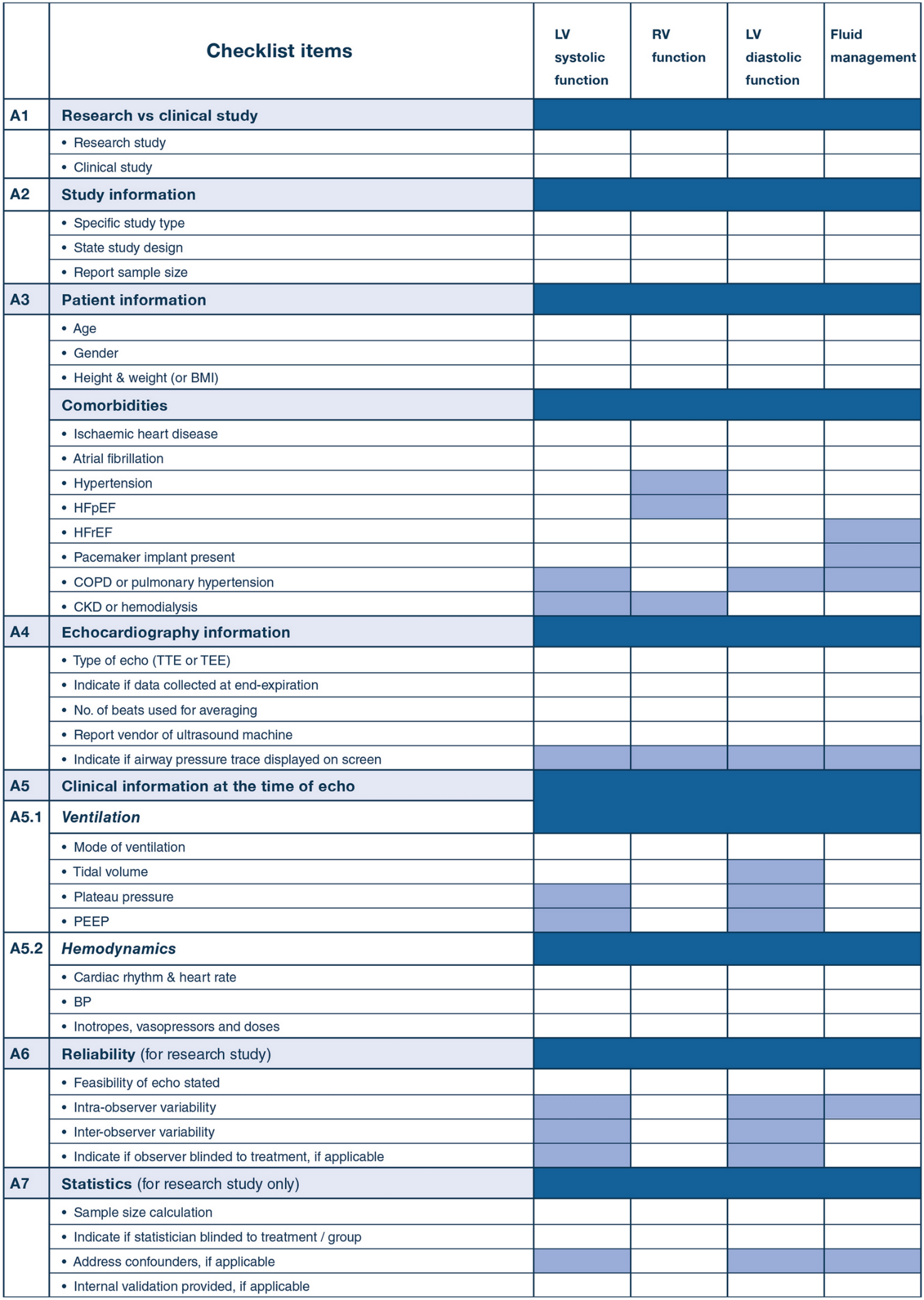


Not Applicable

Not Applicable

Not Applicable

Not Applicable

Not Applicable

Not Applicable


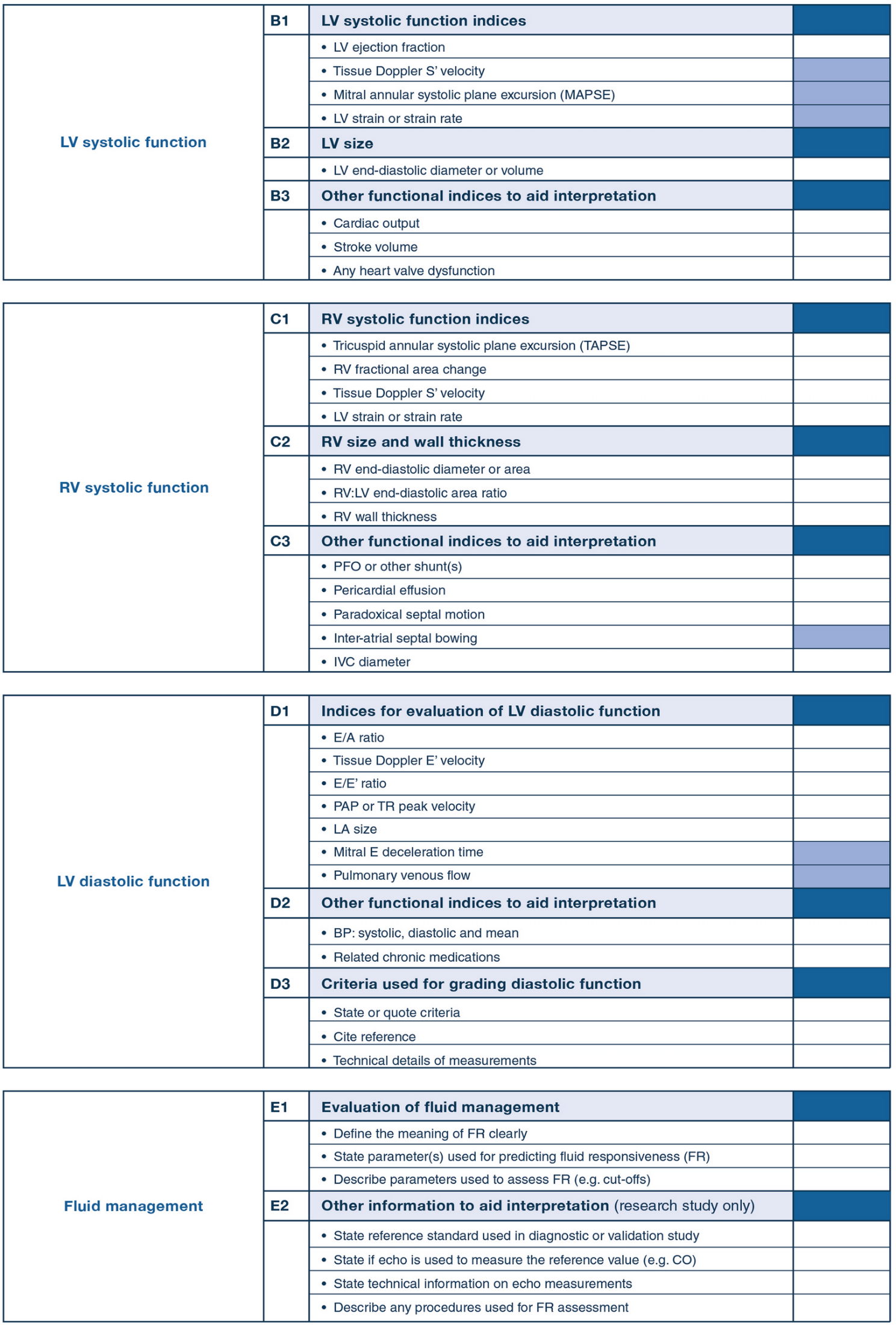

Supplement: Supplementary file 2 — Supplementary file2 (DOCX 1712 KB) [file 40477_2025_1074_MOESM2_ESM.docx]
