# Supplementary material for: Suprasternal ascending or descending aortic velocity peak variability assessment to predict fluid-responsiveness in healthy volunteers: the SADAVA-V pilot prospective study
Source: J Ultrasound. 2025 Sep 18;28(4):931–41. doi: 10.1007/s40477-025-01074-z (PMC12675857; doi:10.1007/s40477-025-01074-z)
Supplement: Supplementary file 3 — Supplementary file3 (DOCX 14 KB) [file 40477_2025_1074_MOESM3_ESM.docx]

|  | SS-V_peak_ (n=65) | AV-V_peak_ (n=64) | p |  | Ascending  SS-V_peak_ (n=22) | Descending  SS-V_peak_ (n=43) |
| --- | --- | --- | --- | --- | --- | --- |
| CO (L/min) | 6.4±1.6 | 6.6±1.6 | 0.068 |  | 6.5±1.1 | 6.4±1.7 |
| SV (ml) | 92.1±18.3 | 94.6±18.4 | 0.179 |  | 95.6±16.7 | 90.1±18.4 |
| HR (bpm) | 71.6±11 | 71.6±10.3 | 0.450 |  | 72.0±9.3 | 71.3±11.9 |
